# Type 1 interferon-inducible gene expression in QuantiFERON Gold TB-positive uveitis: A tool to stratify a high versus low risk of active tuberculosis?

**DOI:** 10.1371/journal.pone.0206073

**Published:** 2018-10-18

**Authors:** Rina La Distia Nora, Ratna Sitompul, Marleen Bakker, Marjan A. Versnel, Sigrid M. A. Swagemakers, Peter J. van der Spek, Made Susiyanti, Lukman Edwar, Soedarman Sjamsoe, Gurmeet Singh, RR Diah Handayani, Aniki Rothova, P. Martin van Hagen, Willem A. Dik

**Affiliations:** 1 Department of Ophthalmology, University of Indonesia & Cipto Mangunkusumo Hospital Kirana, Jakarta, Indonesia; 2 Laboratory of Medical Immunology, Erasmus Medical Center, Rotterdam, the Netherlands; 3 Department of Pulmonary Diseases, Erasmus Medical Center, Rotterdam, the Netherlands; 4 Department of Immunology, Erasmus Medical Center, Rotterdam, the Netherlands; 5 Department of Bioinformatics, Erasmus Medical Center, Rotterdam, the Netherlands; 6 Department of Pathology, Erasmus Medical Center, Rotterdam, the Netherlands; 7 Department of Internal Medicine, Respirology and Critical Illness Division, University of Indonesia & Cipto Mangunkusumo Hospital Kirana, Jakarta, Indonesia; 8 Department of Pulmonology, Persahabatan Hospital, Jakarta, Indonesia; 9 Department of Ophthalmology, Erasmus Medical Center, Rotterdam, the Netherlands; 10 Department of Internal Medicine Section Clinical Immunology, Erasmus Medical Center, Rotterdam, the Netherlands; Colorado State University, UNITED STATES

## Abstract

QuantiFERON-Gold TB (QFT)-positive patients with undetermined cause of uveitis are problematic in terms of whether to diagnose and treat them for tuberculosis (TB). Here, we investigated whether peripheral blood expression of type 1 interferon (IFN)-inducible genes may be of use to stratify QFT-positive patients with uveitis into groups of high versus low risk of having active TB-associated uveitis. We recruited all new uveitis patients in Cipto Mangunkusumo Hospital, Jakarta, Indonesia for one year. We included 12 patients with uveitis and clinically diagnosed active pulmonary TB, 58 QFT-positive patients with uveitis of unknown cause, 10 newly diagnosed sputum-positive active pulmonary TB patients without uveitis and 23 QFT-negative healthy controls. Expression of 35 type 1 IFN-inducible genes was measured in peripheral blood cells from active pulmonary TB patients without uveitis and healthy controls. Differentially expressed genes were identified and used for further clustering analyses of the uveitis groups. A type-1 IFN gene signature score was calculated and the optimal cut-off value for this score to differentiate active pulmonary TB from healthy controls was determined and applied to QFT-positive patients with uveitis of unknown cause. Ten type 1 IFN-inducible genes were differentially expressed between active pulmonary TB and healthy controls. Expression of these 10 genes in QFT-positive patients with uveitis of unknown cause revealed three groups: 1); patients resembling active pulmonary TB, 2); patients resembling healthy controls, and 3); patients displaying an in-between gene expression pattern. A type 1 IFN gene signature score ≥5.61 displayed high sensitivity (100%) and specificity (91%) for identification of active TB. Application of this score to QFT-positive patients with uveitis of unknown cause yielded two groups with expected different likelihood (high vs. low) of having active-TB uveitis, and therefore may be useful in clinical management decisions.

## Introduction

Tuberculosis (TB) is one of the major health problems worldwide. TB-associated uveitis represents a major cause of infectious uveitis in Indonesia and in other countries endemic for TB.[1–5] The diagnosis of TB uveitis is mainly based on microbiological proof of active TB infection in the eye or a positive culture in other organs, most frequently the lungs. However, the diagnosis TB uveitis is challenging in the absence of clinically apparent pulmonary disease because ocular tissue examinations are not readily available and biopsies are troublesome to perform.[6]

The advent of the IFN-γ release assay (IGRA) or QuantiFERON Gold TB (QFT) test enabled the identification of individuals with a prior *Mycobacterium tuberculosis* (*Mtb)* infection. However, although the QFT test provides evidence of an immune response to *Mtb*, it lacks the specificity to distinguish between active and latent TB.[7] Whether QFT-positive uveitis is the result of a direct infection of the retina, an anti-retinal immune response in QFT-positive individuals or a combination of both is unclear.[8] Therefore, using QFT as a diagnostic test for active TB is not feasible in cases suspect of active TB uveitis.

Histopathological studies in patients with uveitis, but without pathological lung findings, documented a *Mtb* infection of the retinal pigment epithelium (RPE). This finding shows that ocular infection may be present even in patients without signs of active systemic TB.[9, 10] Anti-tuberculous therapy (ATT) treatment can be very successful in a part of QFT-positive uveitis of unknown cause, but the results in all QFT-positive patients are not conclusive.[11, 12] Thus, whether a patient has uveitis due to active TB infection, retinal autoimmunity or a combination of both is unclear. Discrimination of active TB infection from latent TB infection-associated uveitis is an important goal for adjusting therapy and optimizing visual outcomes in patients with QFT-positive uveitis.

Recent reports have indicated that active TB is associated with a specific activation pattern of the type 1 IFN signaling cascade.[13–16] Active pulmonary TB can be discriminated from latent TB by the expression of type 1 IFN-inducible genes in immune cells.[13, 15] In this study, we explore whether the expression of type 1 IFN-inducible genes in peripheral blood cells from patients with QFT-positive uveitis can serve as a tool to stratify these patients into groups with a high or low likelihood of having active TB uveitis.

## Patients and methods

### Patients

Patients with uveitis were selected from a prospective uveitis cohort study (June 2014 to May 2015) in which we consecutively recruited 247 patients (after receiving informed consent) with new uveitis referred to the Infection and Immunology Division of the Ophthalmology Department of the Medical Faculty Universitas Indonesia/Cipto Mangunkusumo Hospital, Jakarta, Indonesia. The clinical part of the study has been recently published.[1] For the present study, we excluded 177 of these 247 uveitis patients for the following reasons: 72 had an incomplete screening workup, 29 were human immunodeficiency virus (HIV) positive, 53 were QuantiFERON-Gold TB (QFT)-negative, 2 had an indeterminate QFT value without evidence of clinically active TB, and 21 QFT-positive patients had another established cause of uveitis. In total, we included 12 cases with uveitis with clinically diagnosed active pulmonary TB (of whom two were *Mtb* sputum-positive and 10 were *Mtb* sputum-negative) and 58 patients with uveitis who were QFT-positive without any signs of active TB and in whom no alternative cause for uveitis could be established (Tables 1 and 2). All included patients had active uveitis and only two patients were already receiving ATT at the time of blood sampling.

**Table 1:** General characteristic of the patients.

		Total (n = 103)	Active Pulmonary TB Without uveitis	Uveitis with clinically diagnosed active pulmonary TB	QFT (+) uveitis of unknown cause	Healthy controls	*P* value
			(n = 10)	(n = 12)	(n = 58)	(n = 23)	
**Gender, No.(%)**							0.008
	Male	38 (37)	7 (70)	8 (67)	16 (28)	7 (30)	
	Female	65 (63)	3 (30)	4 (33)	42 (72)	16 (70)	
**Age, mean (SD), years**		40 (15)	41 (16)	42 (17)	46 (13)	31 (9)	0.000
**Microbiologic evidence of Mtb infection, No.(%)**		12 (12)	10 (100)	2 (17)	0 (0)	0 (0)	0.058
**QFT-G value**^**a**^**, median (IQR), IU/ml**		2.8 (0.02–3.7)	2.2 (0.2–5.5)	1.4 (1.0–4.2)	5.0 (2.1–10.4)	0.01 (0.00–0.07)	0.000
**QFT-G positive**^**a**^**, No.(%)**		75 (72)	7 (70)	10 (83)	58 (100)	0 (0)	0.000
**QFT-G negative**^**a**^**, No.(%)**		28 (27)	3 (30)	1 (8)	0 (0)	23 (100)	
**TST > 10 mm**^**b**^**, No.(%)**		49 (83)	NA	10 (100)	39 (84)	NA	0.293
**ATT at the time of blood samping**		2 (2)	0 (0)	2 (17)	0 (0)	NA	NA

Abbreviations: SD, Standard Deviation; *Mtb*, Mycobacterium tuberculosis; IQR, Interquartile Range; QFT, QuantiFERON Gold test; TB, Tuberculosis; TST, Tuberculin Skin Test; NA, Not Applicable.

^a^ QFT-G indeterminate in 1/103 (1%) in suspected tuberculous uveitis with sputum AFB positive

^b^ TST was done in 59 patients due to a temporary unavailability of tuberculin; 10 out of 12 uveitis patients with clinically diagnosed active pulmonary TB and 49 out of 58 QFT (+) unknown cause uveitis patients (in total 59 subjects). TST was not done in all active pulmonary TB patients and healthy controls.

**Table 2:** Ocular characteristics of patients with uveitis*.

Clinical Characteristic		Uveitis with clinically diagnosed active pulmonary TB N(%)	QFT (+) uveitis of unknown cause N(%)
		(Total n = 12)	(Total n = 58)
**Uveitis location**			
	Anterior	1(8)	18(31)
	Intermediate	0(0)	3(5)
	Posterior	5(42)	11(19)
	Panuveitis	6(50)	24(41.5)
	Epi/scleritis	0(0)	2(3.5)
**Laterality**			
	Unilateral	8(67)	33(57)
	Bilateral	4(33)	25(43)
**Duration of uveitis > 1 year**		2 (17)	18(31)
**Visual acuity by Snellen chart (median; IQR)**		0.047; 0.005–0.13	0.045;0.008–2

* All patients had active uveitis though a majority consulted the ophthalmologist already in the late stage of the disease

Abbreviations: TB, Tuberculosis; QFT, QuantiFERON Gold test; IQR, Interquartile Range.

As a positive control group, we additionally included 10 *Mtb* sputum-positive active pulmonary TB patients (HIV negative) without uveitis or a history of ATT from the outpatient clinic of Persahabatan Hospital, East Jakarta, Indonesia. We also included 23 Indonesian healthy controls who were QFT-negative, had no history of uveitis and did not use any medication at the time of the study.

All included patients underwent a full ophthalmic examination. The uveitis classification and grading were performed according to standardized uveitis nomenclature (SUN).[17] The diagnosis and workup were performed as described previously.[1] QFT was performed for all included uveitis patients with the QuantiFERON-Tb Gold (QFT; Cellestis Inc., Carnegie, Australia) test using a positive cut-off value of >0.35 U/mL.[1] The tuberculin skin test (TST) (RT23 SSI-2 T.U/0.1 mL, Statens Serum Institute, Copenhagen, Denmark) was performed in 59/70 uveitis patients due to a temporary unavailability of tuberculin. The TST was considered positive in patients with an induration larger than 10 mm in diameter.[18] The QFT was always performed before the TST, and the TST was not performed in the healthy controls or the active pulmonary TB patients without uveitis.

### Diagnosis of pulmonary TB

Pulmonary TB was diagnosed according to the Tuberculosis Guidelines from the Indonesian Society of Respirology and Indonesian National Guidelines of Tuberculosis Control which were adapted from the World Health Organization (WHO) recommendation: Treatment of Tuberculosis: Guidelines for National Programmes (the algorithm is depicted in S1 Fig).[19–21] Briefly, the diagnosis of active pulmonary TB was based on a clinical examination with the support of microbiological and radiological findings. The review of the chest X-rays and computed tomography (CT) scans was performed by two independent pulmonologists specialized in TB (one from Indonesia (GS) and one from the Netherlands (MB)). The results were classified as being compatible with 1) active TB, 2) prior TB or 3) abnormalities other than TB. The study was approved by the Faculty of Medicine University of Indonesia (FMUI) medical ethics committee, and written informed consent was obtained.

### Whole blood collection

Blood was collected in PAXgene tubes (PreAnalytix, Hombrechtikon, Switzerland) according to the manufacturer’s protocol. The collection was performed at the patient’s first visit to our clinic along with a routine blood draw for the standard uveitis work-up. The collected blood was stored at -80°C for further processing.

### Real-time quantitative PCR

Total RNA was extracted from the whole blood samples using the Blood RNA Extraction Kit (PreAnalytix) and subsequently reverse-transcribed into complementary DNA (cDNA) according to the manufacturer’s instructions. The expression levels of 35 type 1 IFN-inducible genes selected based on previous reports[13–15, 22, 23] (CCL2, CCL7, CX3CR1, CXCL10, DDX58, FCGR1B, GBP1, GBP4, IFI16, IFI27, IFI44, IFI44L, IFIH1, IFIT1, IFIT2, IFIT3, IFITM1, IFNA1, IFNB1, IL15RA, IL1B, IRF7, ISG15, LY6E, MyD88, MxA, OAS1, OAS2, RSAD2, SERPING1, STAT1, TBK1, TLR8, UBE2L6, and USP18) were determined. To calculate the relative expression levels, all the samples were normalized to the expression of the housekeeping gene ABL (ΔC_t_).[24]

### Statistical analysis

Descriptive statistics (mean and standard deviation (SD) or median and interquartile range (IQR)) were used to summarize demographic factors, age, gender, and the TB-related test results (QFT, sputum smear, and TST). Then, the groups were compared using the non-parametric Kruskal-Wallis test or chi-square test. Post hoc analysis was performed with the Bonferroni test.

To identify which of the 35 type 1 IFN-inducible genes differed significantly between the patients with active pulmonary TB without uveitis and the healthy controls, the Mann-Whitney U test was performed. Due to significant age and gender differences between the groups, the analysis was adjusted for age and gender using binary logistic regression.

All statistical analyses were performed in SPSS, and a *P* value <0.05 indicated statistical significance. Unsupervised hierarchical clustering analysis of the identified genes was performed using OmniViz version 6.1.1.13.0 (Instem scientific, Inc.)

#### Type 1 IFN signature score

Individual type 1 IFN-inducible genes were assigned a score (ΔΔC_t_ value) by deducting the ΔC_t_ value of each gene in each subject from the average ΔC_t_ value of that particular gene within the healthy control group and dividing by the standard deviation (SD) of the ΔC_t_ value of that particular gene in the healthy control group. Subsequently, a type 1 IFN signature score was assigned to the total gene set by calculating the sum of the individual gene scores (ΔΔC_t_ values).[23–25]

Receiver operating characteristic (ROC) curve analysis was used to identify the optimal cut-off value of the type 1 IFN signature score associated with active TB. The maximum Youden index (sensitivity + specificity − 1) was used to obtain the optimal cut-off point to discriminate between the active pulmonary TB patients and the healthy controls.

## Results

### Patient groups

The characteristics of the patients and controls are summarized in Tables 1 and 2. A significant difference was found in age and gender between the study groups. The post hoc analysis revealed that the healthy controls were significantly younger than the QFT-positive group with uveitis of unknown origin (p<0.0001). The post hoc analysis of the gender distributions did not reveal any significant differences between the groups.

Among the 12 uveitis patients clinically diagnosed with active pulmonary TB, only 2 had positive *Mtb* staining and PCR (GeneXpert) results in their sputum samples. QFT testing was positive in 10/12 (83%) of these patients. Of the 10 patients with active pulmonary TB but without uveitis, 7/10 (70%) were QFT-positive. Ocular characteristics of patients with uveitis is presented in Table 2.

### Type 1 IFN-inducible gene expression

Among the 35 type 1 IFN-inducible genes, 18 genes differed significantly in their expression levels between the patients with active pulmonary TB without uveitis and the healthy control group (S2 Fig). After adjustment for age and gender, the expression of 10 genes remained significantly different between these two groups. These 10 genes (UBE2L6 (p = 0.004), FCGR1B (p = 0.012), GBP1 (p = 0.013), IL1B (p = 0.015), MYD88 (p = 0.018), TLR8 (p = 0.02), IRF7 (p = 0.028), STAT1 (p = 0.032), SERPING1 (p = 0.036), and IFIT2 (p = 0.037)) were included in the subsequent analyses.

### Cluster analysis of the patient groups and the healthy controls

**Fig 1:**
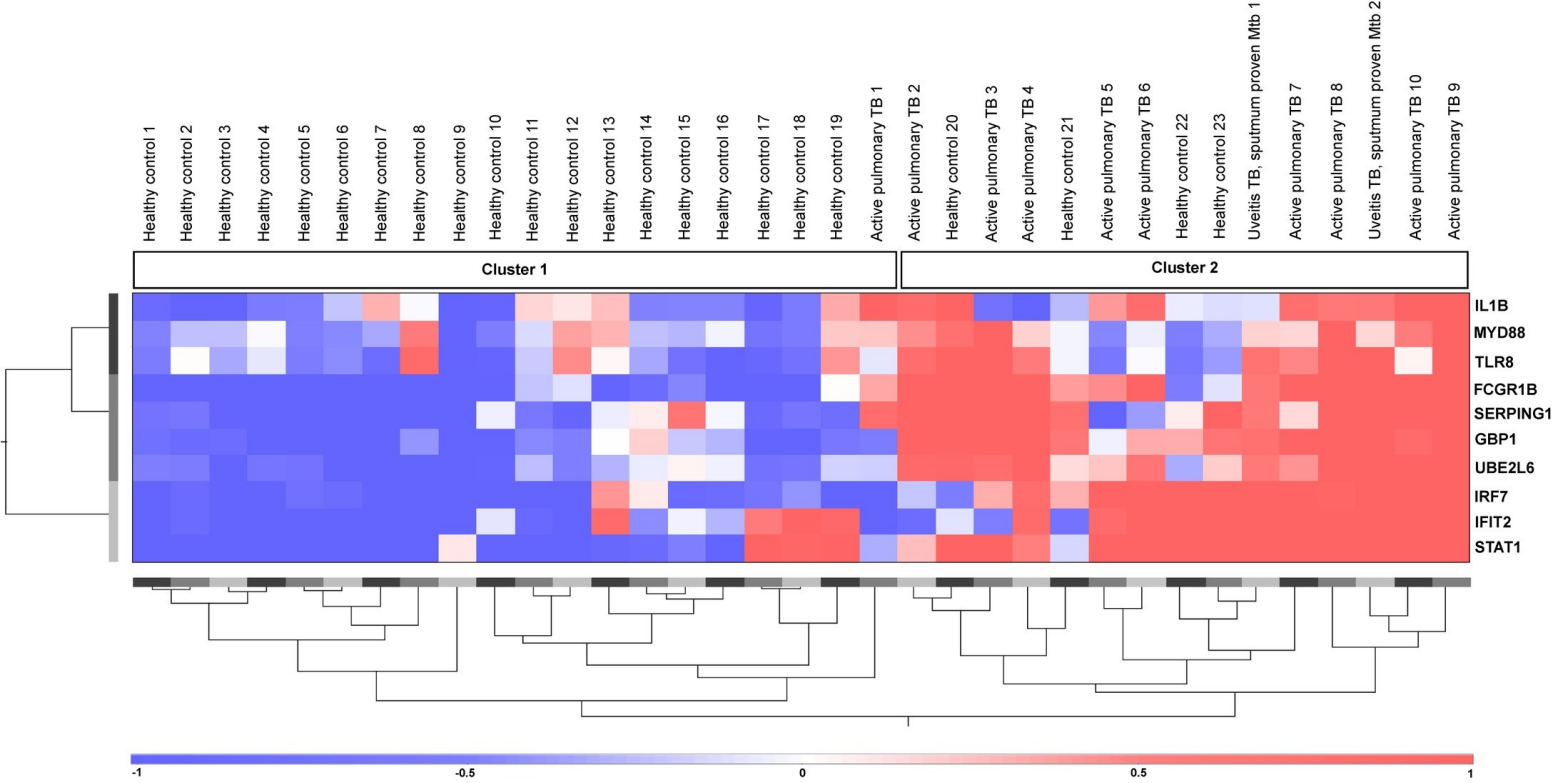
Cluster analysis of patients with uveitis with clinically active, *Mtb* sputum positive, pulmonary TB and patients with active pulmonary TB without uveitis. Unsupervised hierarchical clustering analysis based on 10 type 1 IFN-inducible genes (indicated on the right). Active pulmonary TB subjects indicate the active pulmonary TB patients without uveitis. Uveitis TB subjects indicate uveitis patients with clinically diagnosed active pulmonary TB. Cluster 1 contains the majority of the healthy controls (19/23; 83%) and one active pulmonary TB case without uveitis (1/10; 10%). Cluster 2 contains the majority of the active pulmonary TB cases (9/10; 90%), two uveitis with clinically diagnosed active pulmonary TB with sputum-positive *Mtb* (2/2; 100%) and 4 healthy controls (4/23; 17%). Red indicates increased gene expression levels compared to the geometric mean, and blue indicates decreased gene expression levels compared to the geometric mean. Color intensity correlates with the magnitude of the calculated fold change.

Unsupervised hierarchical clustering of the 10 active pulmonary TB patients without uveitis and 23 healthy controls based on the 10 genes yielded two main clusters. Cluster 1 contained the majority of the healthy controls (19/23; 83%); in contrast, cluster 2 contained the majority of the active pulmonary TB patients without uveitis (9/10; 90%) (S3 Fig). Subsequently, the two *Mtb* sputum-positive uveitis patients with clinically active pulmonary TB were included in this analysis, and both patients were grouped in the cluster that contained 9/10 of the active pulmonary TB patients without uveitis (cluster 2, Fig 1).

**Fig 2:**
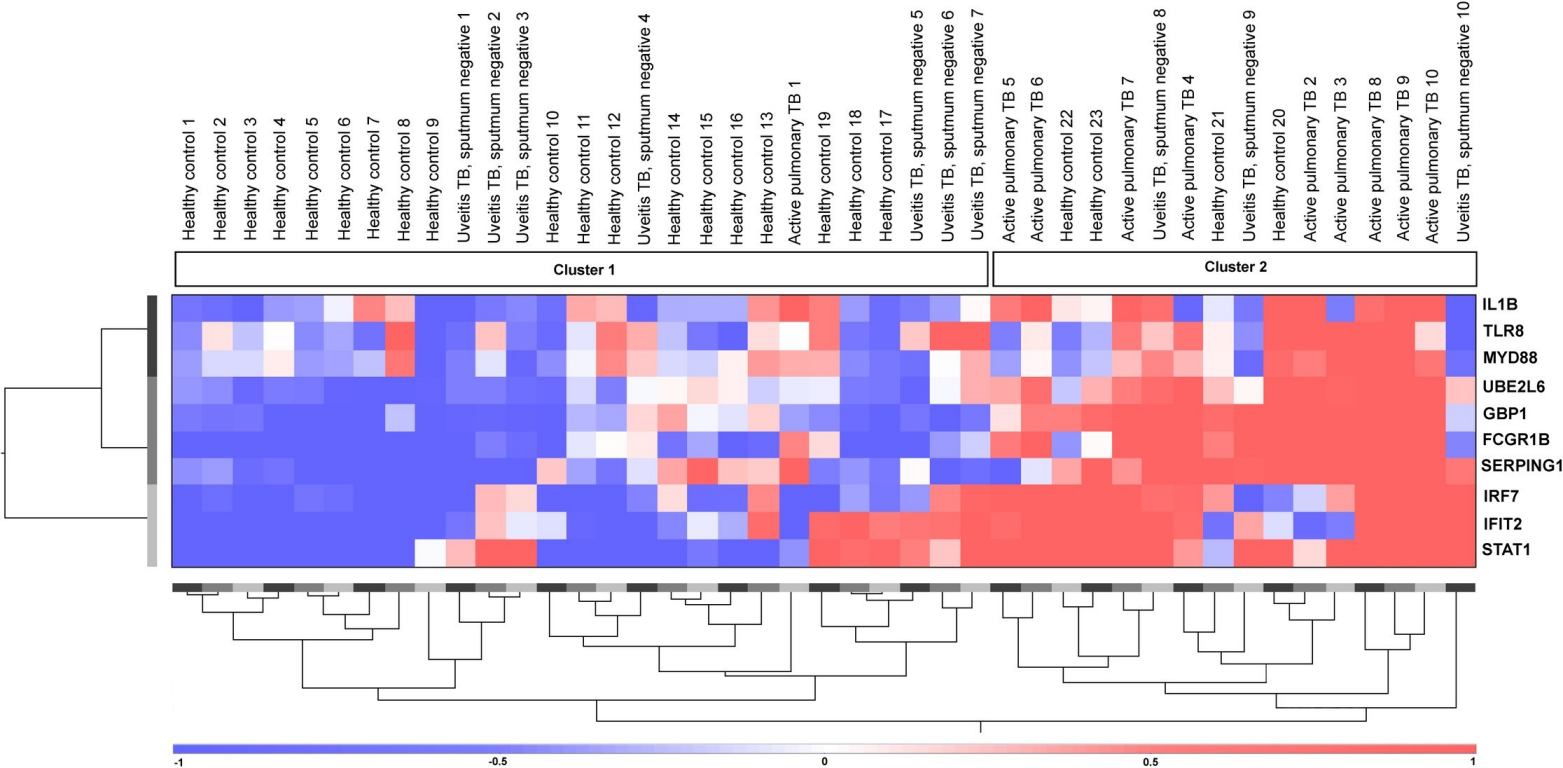
Cluster analysis of patients with uveitis who were sputum *Mtb* negative but were diagnosed with active TB infection on clinical grounds, patients with active pulmonary TB without uveitis and healthy controls. Unsupervised hierarchical clustering analysis based on 10 type 1 IFN-inducible genes (indicated on the right). Active pulmonary TB subjects indicate the active pulmonary TB patients without uveitis. Uveitis TB subjects indicate uveitis patients with clinically diagnosed active pulmonary TB. Cluster 1 contains the majority of the healthy controls (19/23; 83%) and one active pulmonary TB case without uveitis (1/10; 10%). Cluster 2 contains the majority of the active pulmonary TB cases (9/10; 90%) and 4 healthy controls (4/23; 17%). Ten uveitis patients had clinically diagnosed active pulmonary TB infections but negative *Mtb* sputum tests. Only 3 patients (3/10; 30%) were grouped in cluster 2, which primarily contained clustered active pulmonary TB patients. Red indicates increased gene expression levels compared to the geometric mean, and blue indicates decreased gene expression levels compared to the geometric mean. Color intensity correlates with the magnitude of the calculated fold change.

Due to the ability of this 10-gene expression pattern to distinguish the majority of the *Mtb* sputum stain-positive active pulmonary TB patients (both with and without uveitis) from the healthy controls, the same analysis was performed after including the 10 uveitis cases with clinically diagnosed active pulmonary TB who were *Mtb* sputum stain negative. This analysis clustered 7/10 cases (70%) with the majority of the healthy controls (cluster 1), whereas 3 cases (30%) were clustered with the active pulmonary TB cases without uveitis (cluster 2; Fig 2).

**Fig 3:**
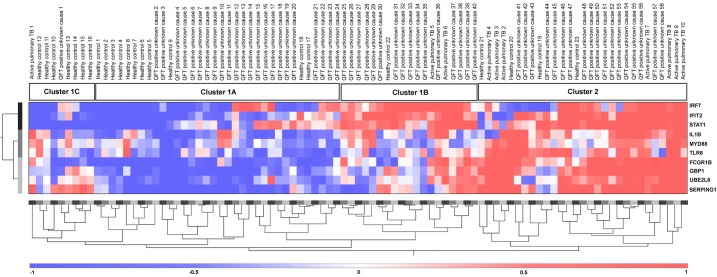
Cluster analysis of QFT-positive patients with uveitis of unknown cause, patients with active pulmonary TB without uveitis and healthy controls. Unsupervised hierarchical clustering analysis based on 10 type 1 IFN-inducible genes (indicated on the right). Active pulmonary TB subjects indicate the active pulmonary TB patients without uveitis. Uveitis TB subjects indicate uveitis patients with clinically diagnosed active pulmonary TB Cluster 1 (A+B+C) contains the majority of the healthy controls (19/23; 83%), cluster 1A contains 11 healthy controls (11/23; 48%) and 23 patients with uveitis of unknown cause were QFT positive (23/58; 40%), cluster 1B contains 1 healthy control (1/23; 4%), 2 patients with active pulmonary TB without uveitis (2/20; 20%) and 16 patients with uveitis of unknown cause were QFT positive (16/58; 28%) and cluster 1C contains 7 healthy controls (7/23; 30%), 1 patient with active pulmonary TB without uveitis (1/10; 10%) and 1patient with uveitis of unknown cause were QFT positive (1/58; 2%). Cluster 2 contains the majority of the active pulmonary TB cases without uveitis (9/10; 90%), 4 healthy controls (4/23; 17%) and 18 patients with uveitis of unknown cause were QFT positive (18/58; 31%). Red indicates increased gene expression levels when compared to the geometric mean, and blue indicates decreased gene expression levels when compared to the geometric mean. Color intensity correlates with the magnitude of the calculated fold change.

Next, the analysis was performed for the 58 QFT-positive patients with uveitis of unknown cause. This analysis revealed that 34/58 patients (59%) were clustered in the vicinity of the majority of the active pulmonary TB patients without uveitis (clusters 2 and 1B, Fig 3), whereas 23/58 (37%) were clustered close to the majority of the healthy controls (cluster 1A, Fig 3). Additionally, 1/58 patients (4%) was grouped together with 7 healthy controls and 1 active TB without uveitis case (cluster 1C, Fig 3). However, the gene expression pattern in cluster 1B was more similar to that of cluster 2, which contained the majority of the active pulmonary TB cases.

### Type 1 IFN gene signature score

**Fig 4:**
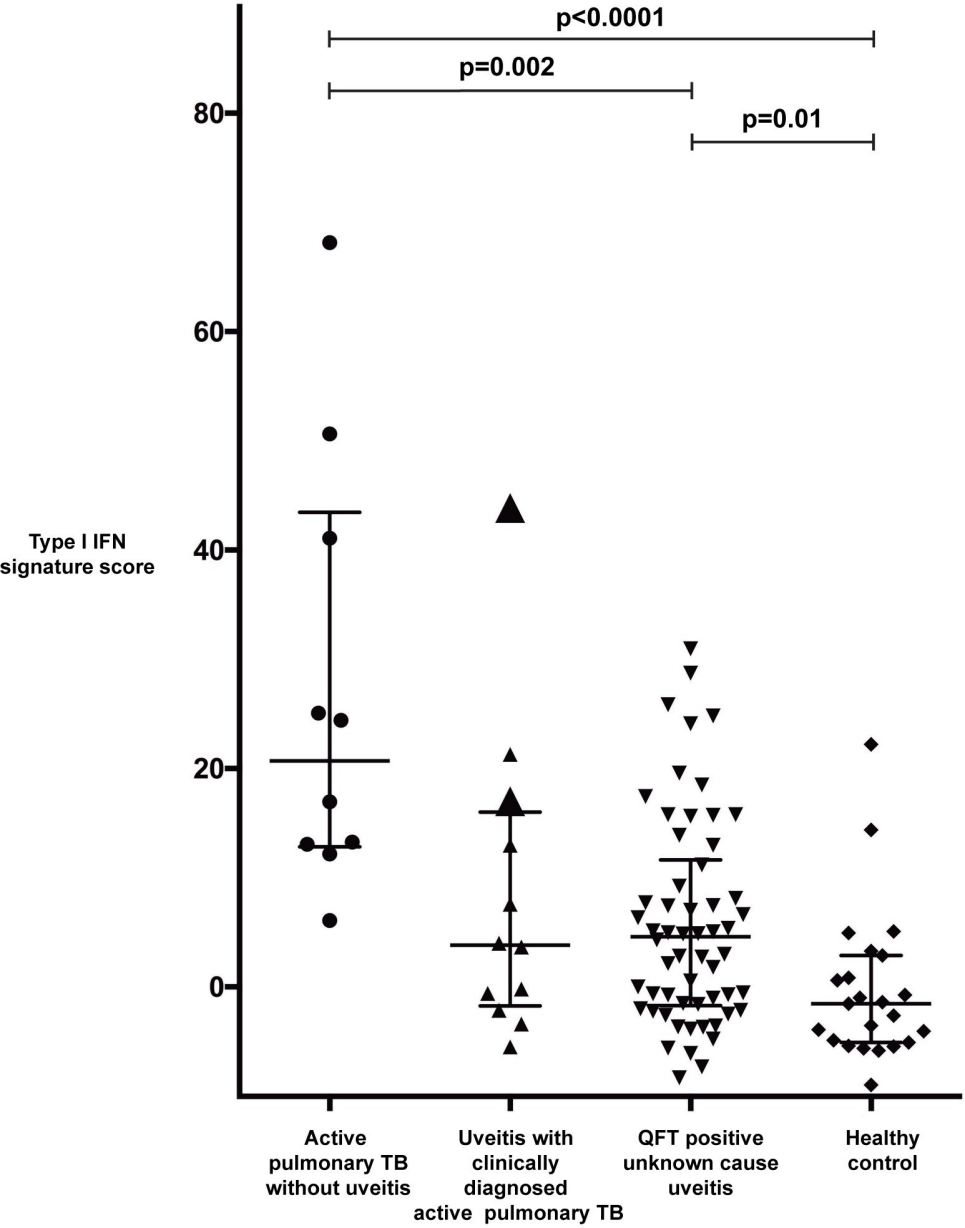
Scatter plot of type 1 IFN signature scores for the four study groups. A type 1 IFN gene signature score was calculated for every individual patient sample based on the expression of the 10-gene set. Each point represents one individual. Horizontal bars indicate the median value within a group. Enlarged triangles within the TB uveitis group represent the two individuals within that group with a sputum stain positive for *Mtb*. Statistical significance was assessed using the Kruskal-Wallis test, and subgroup analyses were performed with Dunn’s multiple comparison test.

**Fig 5:**
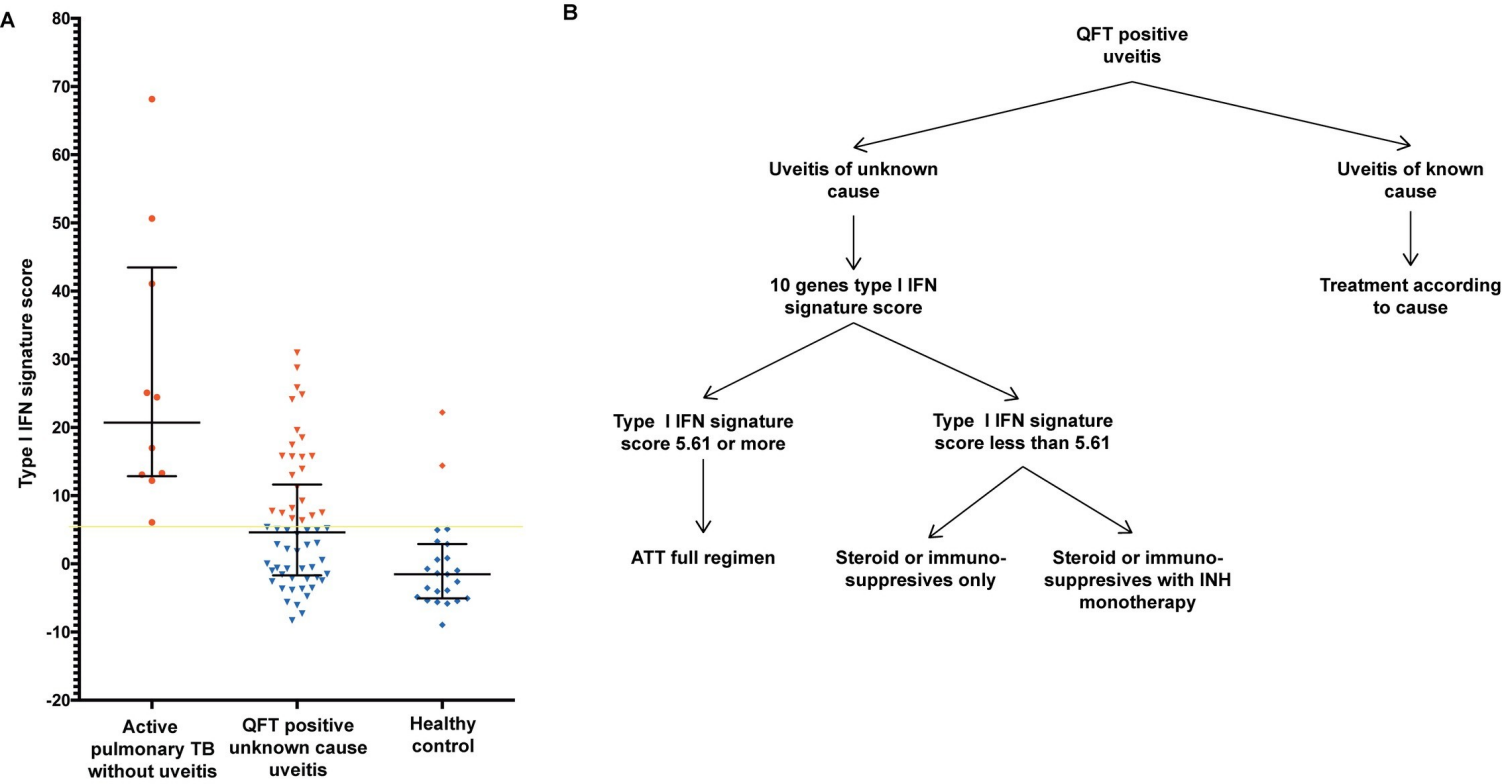
Proposed algorithm for the diagnostic work-up of QFT-positive uveitis. A. Scatter plot of type 1 IFN signature scores for QFT-positive patients with uveitis of unknown cause, patients with active pulmonary TB without uveitis and healthy controls. Each point represents one individual. Horizontal bars indicate the median value within a group. Yellow horizontal bar marks the cut off value of 5.61 for a positive versus a negative type 1 IFN gene signature score. Therefore, symbols indicated in red represent type1 IFN gene signature score positive individuals and blue symbols indicate type1 IFN gene signature score negative individuals. B. Algorithm for the management of QFT-positive uveitis patients. QFT-positive patients with uveitis of known causes should be treated appropriately for that cause. In QFT-positive patients with uveitis of unknown causes, the 10-gene type 1 IFN score can be calculated. When the score is 5.61 or more, we recommend to give the patient a full ATT regimen and the necessary anti-inflammatory treatment regimen. When the score is less than 5.61, the patient can defer ATT and can undergo anti-inflammatory treatment with or without an Isoniazid (INH) prophylaxis regimen with strict observation.

Based on the 10-gene expression pattern, a type 1 IFN signature score was calculated for each individual included in the study groups. This calculation revealed a significant difference between the active pulmonary TB patients without uveitis and the healthy controls (p<0.0001; Fig 4). The type 1 IFN signature score of QFT-positive patients with uveitis of unknown cause had significantly lower scores than the active pulmonary TB patients without uveitis (*P* value 0.002) but significantly higher scores than the healthy controls (*P* value 0.01; Fig 4). In the patients with uveitis and clinically diagnosed active pulmonary TB, the two sputum-smear-positive TB uveitis patients and three other cases revealed type 1 IFN signature scores that were comparable to those seen in the active pulmonary TB without uveitis group. Furthermore, in the QFT-positive patients with uveitis of unknown cause, the type 1 IFN signature scores varied, with a range from scores comparable to those seen in the active pulmonary TB (without uveitis) group to scores overlapping those seen in the healthy control group (Fig 4). Further, we determined a type 1 IFN signature score cut-off value using ROC curve analysis that yielded an area under the curve (AUC) of 0.961 with a *P* value <0.00001 (S4A Fig); a cut-off value ≥5.61 displayed the highest sensitivity (100%), specificity (91%) and Youden index (0.913) for the identification of active TB (S4B Fig and Fig 5A).

## Discussion

In this study, we demonstrate that a blood transcriptional signature of 10 type 1 IFN-inducible genes differentiates QFT-positive uveitis patients into distinct different groups.

Type 1 IFN (IFNα/β)-inducible gene expression signatures in peripheral blood cells have consistently been reported as a potential biomarker for active pulmonary TB compared with signatures in healthy controls or patients with other diseases.[13] Berry *et al*. first reported the association between active pulmonary TB and the expression of numerous type 1 IFN-inducible genes in peripheral blood cells in regions with both an intermediate TB burden (London, UK) and a high TB burden (South Africa)[13]. Importantly, these type 1 IFN-inducible gene transcripts differed from those observed in systemic autoimmune diseases, including systemic lupus erythematosus (SLE), which is among a group of systemic autoimmune diseases that contains subgroups of patients displaying a type 1 IFN-inducible gene expression pattern.[13, 22, 26, 27] Several other studies have corroborated the use of type 1 IFN-inducible gene expression in the peripheral blood to distinguish active from latent TB infection.[14–16, 28, 29] Moreover, a rapid normalization of the type 1 IFN gene expression pattern upon successful ATT was reported.[13, 15] Together, these observations underscore the role of type 1 IFN signaling in the pathogenesis of TB and the potential usefulness of the peripheral blood type 1 IFN transcriptome as a biomarker and monitoring tool in TB diagnostics and management. However, no studies of type 1 IFN-inducible blood transcriptome have been conducted in uveitis cases in relation to TB infection.

In our study, we identified a peripheral blood cell transcriptome consisting of 10 type 1 IFN-inducible genes that were strongly associated with active pulmonary TB without uveitis. When a type 1 IFN signature score was applied to this 10-gene set, a score ≥5.61 displayed the optimal sensitivity and specificity for distinguishing active pulmonary (*Mtb* sputum smear-positive) TB patients without uveitis from healthy controls. In line with this result, the two TB uveitis cases diagnosed with active pulmonary TB and having positive *Mtb* sputum smear displayed a type 1 IFN signature score >5.61. This finding indicates that microbiologically proven active pulmonary TB with or without uveitis is associated with high expression of type 1 IFN-inducible genes. However, several additional uveitis cases, who were QFT-positive, displayed a positive type 1 IFN signature despite being *Mtb* sputum negative.

Over-diagnosis of uveitis TB due to the lack of a gold standard diagnostic test (i.e., laboratory and radiological investigations) is a recognized problem that may result in overzealous treatment of uveitis with ATT.[30] Within the uveitis group diagnosed with pulmonary TB on clinical grounds solely, we found 7 cases with a type 1 IFN signature score <5.61. In these cases, the uveitis was probably not related to active TB. Importantly, the type 1 IFN signature scores revealed two subgroups within the QFT-positive patients with uveitis of unknown cause. We propose that QFT-positive patients with uveitis of unknown cause and a type 1 IFN signature score <5.61 are unlikely to have uveitis related to active TB infection. This conclusion is supported by the high concordance within the set of 10 type 1 IFN-inducible genes identified in our study and the type-1 IFN inducible genes identified in other studies that did compare active with latent TB.[16] Furthermore, this finding is in agreement with other studies that compared patients with active and latent TB and reported a rapid decline in peripheral blood cell expression of type 1 IFN-inducible genes in active TB patients treated with a full ATT regimen.[13, 15] Consequently, we expect that patients within the QFT-positive group with uveitis of unknown cause who display a positive type 1 IFN signature score (≥5.61) will have a higher likelihood of having uveitis due to active TB infection and beneficial reaction to ATT. Our series, however, lacks the results of type 1 IFN signature in QFT-positive patients without uveitis. Nevertheless, we propose that measurement of a peripheral blood type 1 IFN gene signature score can aid to the diagnostic workup and choice of treatment in patients with QFT-positive uveitis, as is indicated in Fig 5B.

Elevated expression of type 1 IFN-inducible genes in peripheral blood cells is not specific to active TB disease. Type 1 IFN gene signatures are also associated with systemic autoimmune diseases, such as in Sjögren’s syndrome cases (~55%), systemic lupus erythematosus (~50%), and systemic sclerosis (~30%), and are also present in healthy control subjects (~5%).[22, 26, 27] However, differential expression of type 1 IFN-inducible genes between TB and systemic autoimmune diseases is clear. MxA is a type 1 IFN-inducible gene that encodes an important mediator of the early innate immune defense against viruses.[23] Elevated MxA expression is part of the peripheral blood type 1 IFN signature in systemic autoimmune disease patients.[23] Yet in our study elevated MxA gene expression was not found in peripheral blood from patients with active pulmonary TB compared to healthy control subjects (S2 Fig). Therefore, our data support differential involvement of type 1 IFN-inducible genes among different diseases associated with type 1 IFN activity.[13]

In autoimmune disease activated plasmacytoid dendritic cells (pDCs) are thought to be the most dominant producers of type 1 IFN and thus inducers of the type 1 IFN gene signature, although other type 1 IFN producing cells are not excluded.[31, 32] There is data supporting involvement of pDCs in TB pathophysiology and pDCs may be a source of type 1 IFN in TB.[33–38] Yet, the relation between pDCs, type1 IFN and TB pathophysiology remains hardly studied so far and needs to be further elucidated. Moreover, other celtypes, including macrophages and epithelial cells may represent a source of type 1 IFN in TB.[39] And more recently we demonstrated that *Mtb* infected retinal pigment epithelial cells are also strong producers of type 1 IFN and as such may be relevant to TB-associated uveitis.[40]

Our study was of exploratory nature and set up to identify whether a peripheral type 1 IFN gene signature could stratify *Mtb*-associated uveitis patients, either amongst patients with active TB disease or healthy controls. This is especially relevant for the diagnostic problematic group of patients that are QFT-positive and have uveitis of unknown cause, and subgroups (positive and negative type 1 IFN signature) could indeed be recognized, and as such might be qualified as high or low risk of having uveitis in relation to active TB. We did not include QFT-negative uveitis patients in our study as they are never suspected of TB-associated uveitis. However, it can be anticipated that a proportion of QFT-negative uveitis cases will be associated with a positive type-1 IFN gene signature, for instance in case of uveitis in association with autoimmune disease or toxoplasmosis.

In conclusion, we identified a blood transcriptional signature of 10 type 1 IFN-inducible genes highly expressed in active pulmonary TB patients. Although further validation studies are required, our data suggest that this peripheral blood type 1 IFN gene signature has the potential to stratify patients with suspected active TB infection-associated uveitis into groups with either a low or high risk of having uveitis due to TB. We expect that this stratification forms a constructive basis for future diagnostic and treatment studies in QFT-positive patients with uveitis.

## Supporting information

S1 Fig Indonesian Society of Respirology (ISR) TB diagnostic guideline. Scheme depicting the TB diagnostic algorithm in Indonesia. The guideline starts with evaluation of clinical signs suggestive for tuberculosis (TB). In patients with signs suggestive of pulmonary TB infection, an acid-fast staining examination from the sputum is required and a positive result leads to diagnosis of active pulmonary TB. If the culture examinations from the sputum are negative, radiologic imaging is reviewed and classified. When imaging is considered to exhibit signs of active pulmonary TB, the patient is diagnosed with active pulmonary TB. Therefore, active pulmonary TB will be either acid-fast bacillus (AFB) positive or AFB negative. The diagnosis of AFB-positive active pulmonary TB is based on positive culture or positive GeneXpert outcomes, and AFB-negative TB is based on radiologic signs of active pulmonary TB and clinical improvement after anti-tuberculosis antibiotic treatment.
TB: tuberculosis, AFB: acid-fast bacillus, GeneXpert: a molecular test that detects bacterial DNA from *Mycobacterium tuberculosis*, PCR: polymerase chain reaction, CXR: chest X-ray, TST: tuberculin skin test.
(TIF)

S2 Fig Type 1 IFN-inducible genes with significant differences in expression between active pulmonary TB patients without uveitis and healthy controls. Of a total of 35 type 1 IFN-inducible genes measured in the peripheral blood, 18 genes differed significantly in their expression between the active pulmonary TB without uveitis patients and the healthy controls without correction for age differences between the groups. Statistical significance was assessed using the Mann-Whitney U test. **** *P* value <0.0001, *** *P* value <0.001, ** *P* value <0.01, * *P* value <0.05.
(TIF)

S3 Fig Cluster analysis of active pulmonary TB patients without uveitis and healthy controls based on type 1 IFN-inducible gene expression patterns. Unsupervised hierarchical clustering analysis based on 10 type 1 IFN-inducible genes (indicated on the right). Active pulmonary TB subjects indicate the active pulmonary TB patients without uveitis. Cluster 1 contains the majority of the healthy controls (19/23; 83%) and one active pulmonary TB case without uveitis (1/10; 10%). Cluster 2 contains the majority of the active pulmonary TB cases without uveitis (9/10; 90%) and 4 healthy controls (4/23; 17%). Red indicates increased gene expression levels when compared to the geometric mean, and blue indicates decreased gene expression levels when compared to the geometric mean. Color intensity correlates with the magnitude of the calculated fold change.
(TIF)

S4 Fig ROC curve analysis and Youden Index calculation for determining the optimal cut-off value of type 1 IFN signature score. **A.** The ROC curve analysis and area under the curve (AUC) **B.** Youden index analyses indicate that a type 1 IFN signature score cut-off value ≥5.61 represents the optimal cut-off value for distinguishing active pulmonary TB patients without uveitis from healthy controls with 100% sensitivity, 91% specificity and a Youden index of 0.913.
(TIF)
