# Supplementary figures and images for: Type 1 interferon-inducible gene expression in QuantiFERON Gold TB-positive uveitis: A tool to stratify a high versus low risk of active tuberculosis?
Source: PLoS One. 2018 Oct 18;13(10):e0206073. doi: 10.1371/journal.pone.0206073 (PMC6193765; doi:10.1371/journal.pone.0206073)

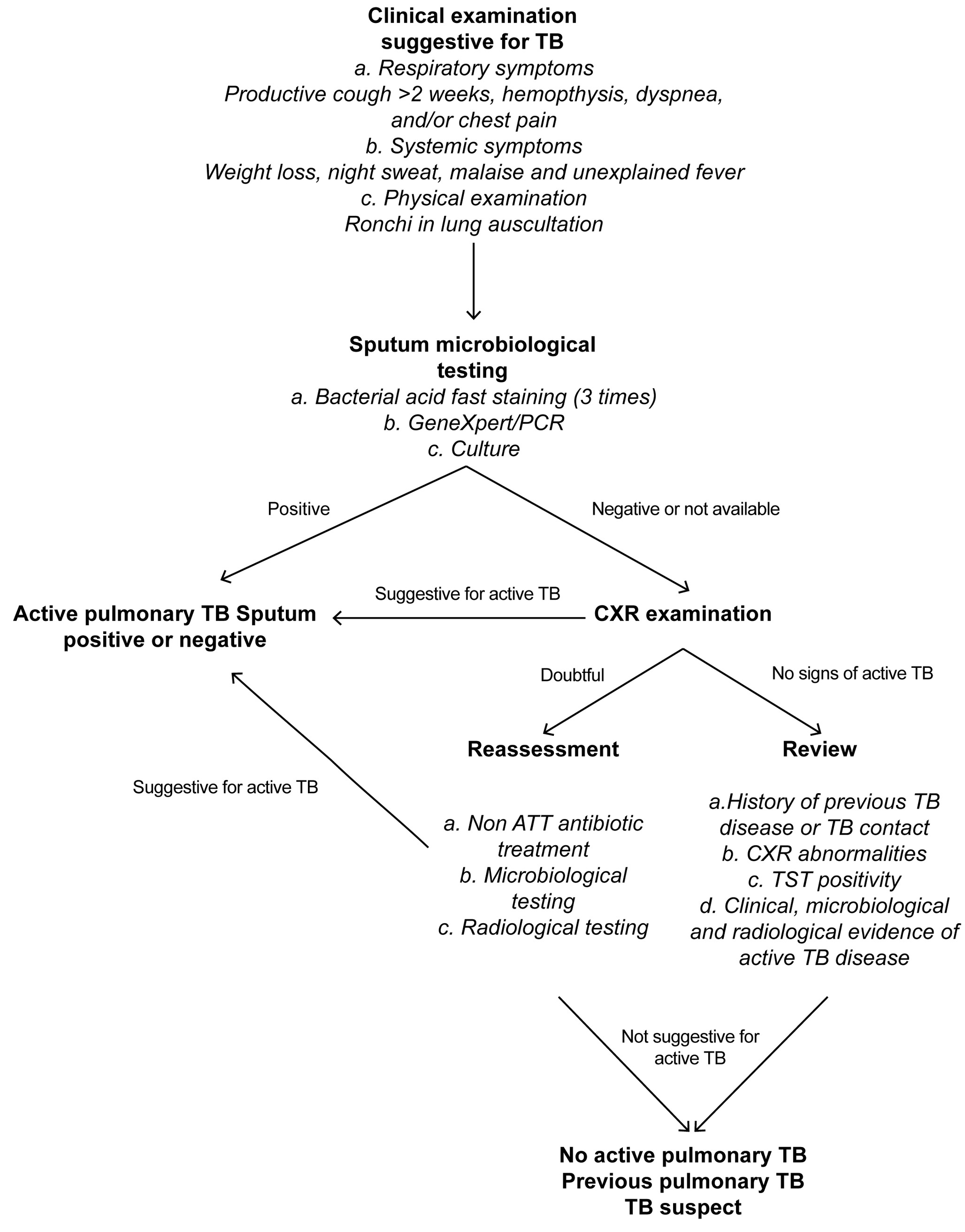

Supplement: S1 Fig — Scheme depicting the TB diagnostic algorithm in Indonesia. The guideline starts with evaluation of clinical signs suggestive for tuberculosis (TB). In patients with signs suggestive of pulmonary TB infection, an acid-fast staining examination from the sputum is required and a positive result leads to diagnosis of active pulmonary TB. If the culture examinations from the sputum are negative, radiologic imaging is reviewed and classified. When imaging is considered to exhibit signs of active pulmonary TB, the patient is diagnosed with active pulmonary TB. Therefore, active pulmonary TB will be either acid-fast bacillus (AFB) positive or AFB negative. The diagnosis of AFB-positive active pulmonary TB is based on positive culture or positive GeneXpert outcomes, and AFB-negative TB is based on radiologic signs of active pulmonary TB and clinical improvement after anti-tuberculosis antibiotic treatment. TB: tuberculosis, AFB: acid-fast bacillus, GeneXpert: a molecular test that detects bacterial DNA from Mycobacterium tuberculosis, PCR: polymerase chain reaction, CXR: chest X-ray, TST: tuberculin skin test. (TIF) [file pone.0206073.s001.tif]

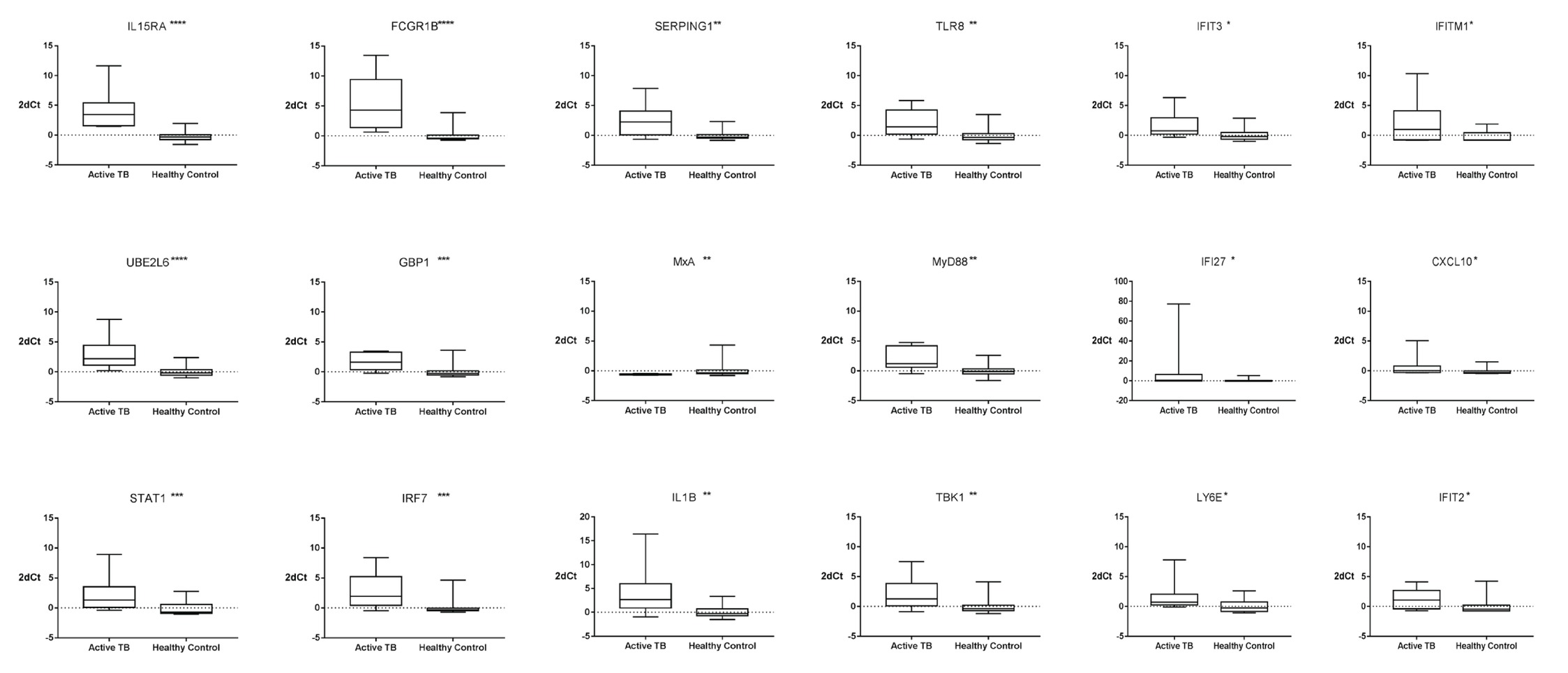

Supplement: S2 Fig — Of a total of 35 type 1 IFN-inducible genes measured in the peripheral blood, 18 genes differed significantly in their expression between the active pulmonary TB without uveitis patients and the healthy controls without correction for age differences between the groups. Statistical significance was assessed using the Mann-Whitney U test. **** P value <0.0001, *** P value <0.001, ** P value <0.01, * P value <0.05. (TIF) [file pone.0206073.s002.tif]

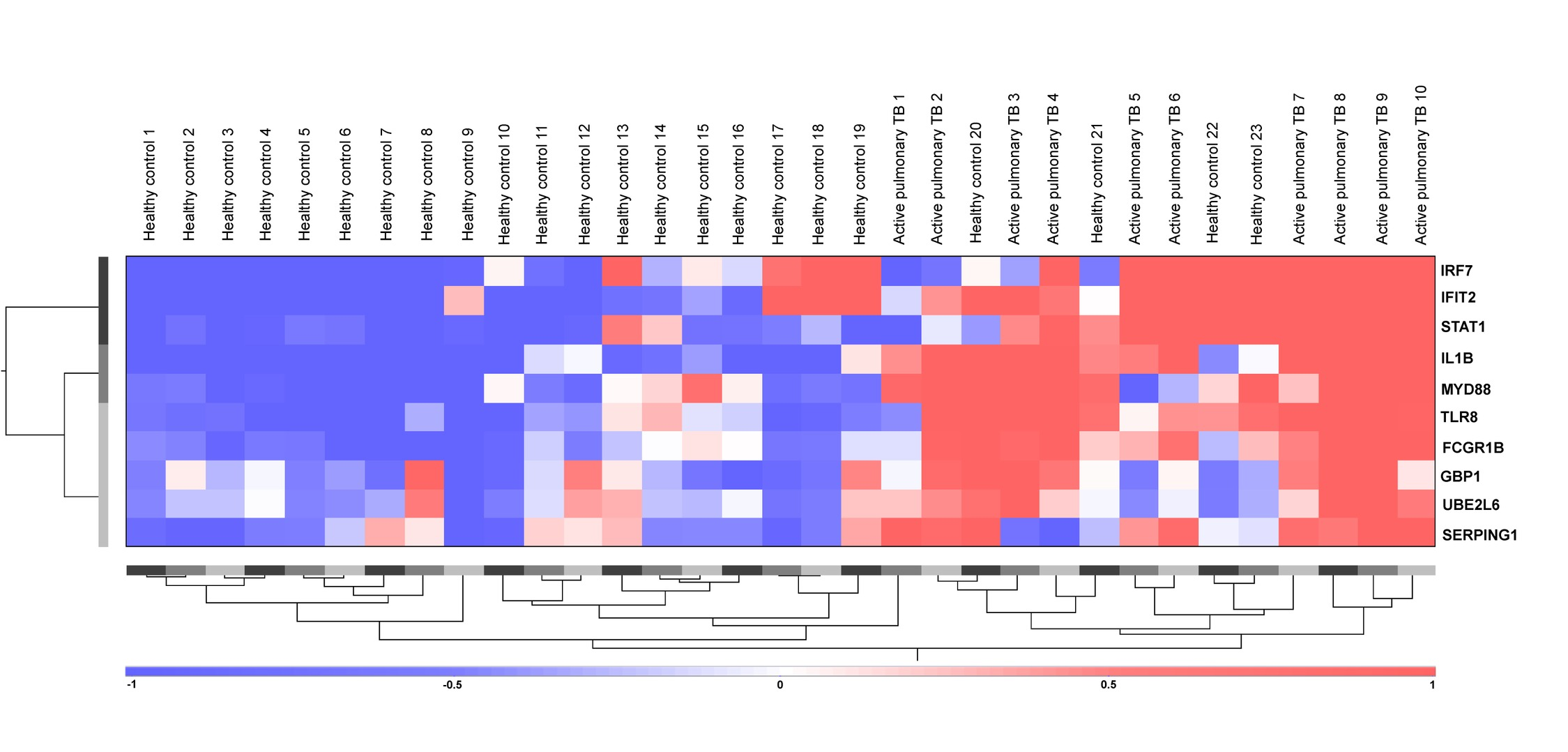

Supplement: S3 Fig — Unsupervised hierarchical clustering analysis based on 10 type 1 IFN-inducible genes (indicated on the right). Active pulmonary TB subjects indicate the active pulmonary TB patients without uveitis. Cluster 1 contains the majority of the healthy controls (19/23; 83%) and one active pulmonary TB case without uveitis (1/10; 10%). Cluster 2 contains the majority of the active pulmonary TB cases without uveitis (9/10; 90%) and 4 healthy controls (4/23; 17%). Red indicates increased gene expression levels when compared to the geometric mean, and blue indicates decreased gene expression levels when compared to the geometric mean. Color intensity correlates with the magnitude of the calculated fold change. (TIF) [file pone.0206073.s003.tif]

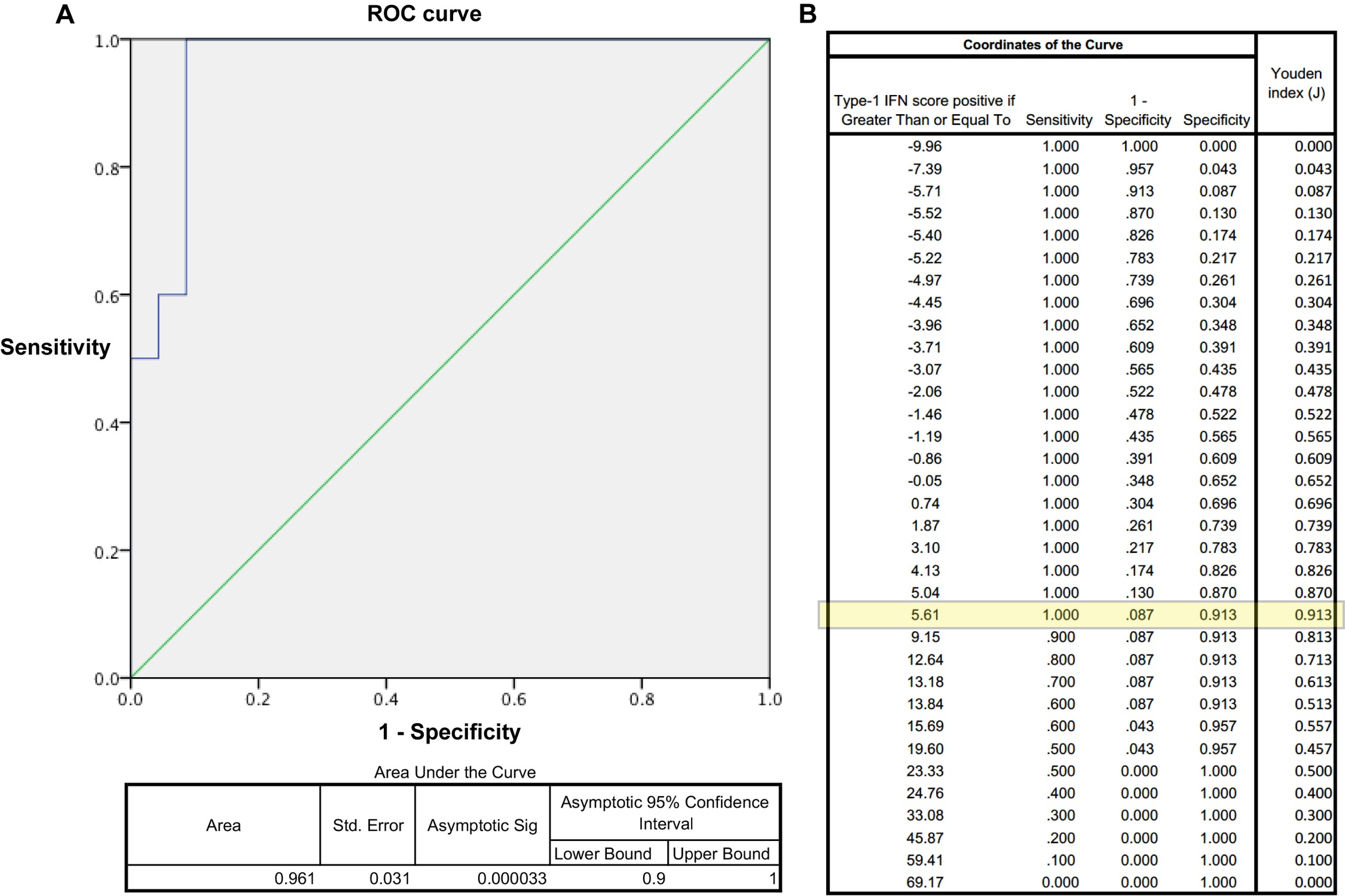

Supplement: S4 Fig — A. The ROC curve analysis and area under the curve (AUC) B. Youden index analyses indicate that a type 1 IFN signature score cut-off value ≥5.61 represents the optimal cut-off value for distinguishing active pulmonary TB patients without uveitis from healthy controls with 100% sensitivity, 91% specificity and a Youden index of 0.913. (TIF) [file pone.0206073.s004.tif]
